# Severe acute exacerbation of chronic hepatitis B during pegylated interferon treatment and early intervention with corticosteroid

**DOI:** 10.1186/1743-422X-9-136

**Published:** 2012-07-24

**Authors:** Qing Mao, Hui-Yan Zhang, Jian-Ping You, Xu-Qing Zhang

**Affiliations:** 1 Department of Infectious Diseases, Southwest Hospital, Third Military Medical University, Chongqing, 400038, People's Republic of China

**Keywords:** Chronic hepatitis B, Acute-on-chronic liver failure, Severe acute exacerbation, Corticosteroid, Pegylated interferon

## Abstract

Severe acute exacerbation or liver failure induced by standard interferon-α(IFN-α) therapy had been reported to occur in few patients with chronic hepatitis B. However, no report showed that pegylated interferon-α therapy was able to induce severe acute exacerbation of chronic hepatitis B. Here, we describe three patients with severe acute exacerbation of chronic hepatitis B during pegylated interferon-α2a (Pegasys) treatment. One patient progressed into acute-on-chronic liver failure (ACLF) at the second week of Pegasys treatment. Two patients progressed into acute-on-chronic pre-liver failure (pre-ACLF) at the second and eighth week of Pegasys treatment, respectively. Three patients recovered after early combined intervention with corticosteroid and lamivudine. Our data indicated that there was a risk of severe acute exacerbation among patients with chronic hepatitis B during receiving Pegasys treatment. Importantly, early combined intervention with corticosteroid and lamivudine should be introduced to prevent the disease progression and improve their prognosis once severe acute exacerbation was diagnosed.

## Introduction

It is estimated that over 350 million people worldwide are chronically infected with hepatitis B virus (HBV). Chronic hepatitis B is one of the most important causes of cirrhosis and hepatocellular carcinoma [1]. Injected interferons (standard interferon-α and pegylated interferon-α) are approved to treat chronic hepatitis B in many countries, and have been confirmed to be effective in preventing the disease progression of chronic hepatitis B. Acute exacerbation of chronic hepatitis B is not uncommon during interferon-α(IFN-α) therapy because that IFN-αexerts a variety of immuno-modulatory effects [2]. However, there were very few reports about severe acute exacerbation of chronic hepatitis B during IFN-α therapy [3,4]. From January 2008 to December 2010, 254 patients with chronic hepatitis B received Pegylated interferon-α2a (Pegasys) therapy in our department. During Pegasys therapy, alanine aminotransferase (ALT) flare was observed in 25.2% (64/254) patients, including 9.4% (24/254) patients with serum ALT ≥ 420 IU/L (normal range 0–42 IU/L) and serum total bilirubin (T-Bil) ≤ 51.3 μmol/L (normal range 6–17.1 μmol/L), and 1.2% (3/254)patients with severe acute exacerbation (serum ALT ≥ 420 IU/L and T-Bil >51.3 μmol/L). In addition, severe acute exacerbation of chronic hepatitis B having a potential for progression to acute on chronic liver failure (ACLF) or acute on chronic pre-liver failure (pre-ACLF) with extremely high mortality, so it is highly important how to prevent the disease progression [5,6]. Here, we introduced three patients with severe acute exacerbation of chronic hepatitis B during Pegasys treatment, including two patients with pre-ACLF and one patient with ACLF. Three patients were successfully treated with early intervention of corticosteroid and lamivudine.

## Case report

### Patients

Three chinese male patients were hospitalized in the Department of Infectious Diseases, Southwest Hospital, Third Military Medical University, China, between January 2009 and October 2010. Serologic tests for hepatitis A virus (HAV), hepatitis C virus (HCV), hepatitis D virus (HDV), hepatitis E virus (HEV),cytomegalovirus (CMV), Epstein-Barr virus (EBV), and human immunodeficiency virus (HIV), and immunologic tests for liver and kidney microsomal, mitochondrial, smooth muscle, and nuclear antibodies were negative in three patients before and after receiving Pegasys therapy. Three patients were excluded to have cirrhosis by abdominal ultrasound, abdominal computed tomography scan, and Fibroscan before receiving Pegasys therapy. They did not receive any drugs other than Pegasys or alcoholism before acute exacerbation of chronic hepatitis B. Clinical characteristics of three patients with chronic hepatitis B at the baseline of interferon treatment were shown in Table 1.

**Table 1:** Clinical characteristics at the baseline of Pegasys treatment

	Case 1	Case 2	Case 3
**Age (yr)**	**23**	**54**	**42**
**Course of disease (yr)**	**6**	**2**	**17**
**HBV genotype**	**B**	**C**	**B**
**HBeAg**	**+**	**-**	**-**
**Anti-HBe**	**-**	**+**	**+**
**HBV DNA (Lc/ml**	**7.78**	**6.90**	**6.99**
**WBC^x^10/L**	**7.5**	**5.89**	**5.69**
**Hb (g/L)**	**156**	**137**	**152**
**Plt(^x^10/L**	**135**	**112**	**101**
**ALT (lU/L)**	**333**	**71**	**304**
**AST (lU/L)**	**215**	**76**	**149**
**AST/ALT ratio**	**0.65**	**1.07**	**0.49**
**ALP (lU/L)**	**197**	**123**	**94**
**y-GTP(lU/L**	**45**	**27**	**44**
**Total bilirubin(umol/L**	**13.9**	**16.9**	**30.9**
**Prothrombin activity (%)**	**105**	**100**	**104**
**AFP (ng.ml)**	**3.31**	**5.6**	**4.29**
**Creatinine (**μmol/L)	**59**	**79**	**81**
**T3 free (pmol/L)**	**4.1**	**3.9**	**3.6**
**T4 free (pmol/L)**	**14.3**	**11.3**	**12.7**
**TSH (mlU/L**	**2.1**	**1.2**	**1.4**
**Fibroscan (kPa)**	**4.3**	**5.1**	**5.7**

yr: year; Course of disease: interval between the first time when HBsAg-positive was detected and the date before treatment; HBV: hepatitis B virus; HBeAg: hepatitis B e antigen; LC/ml: Log copies/ml; WBC: white blood cell; Hb: hemoglobin; Plt: platelet; ALT: alanine aminotransferase; AST: asparate aminotransferase; ALP: alkaline phosphatase; γ-GTP: gamma glutamil transpeptidase; AFP: alpha fetoprotein; T3 and T4: thyroxin 3 and 4; TSH: thyroid-stimulating hormone.

### Severe acute exacerbation of hepatitis B during pegasys treatment

Severe acute exacerbation of chronic hepatitis B was observed in two patients at the second week of Pegasys treatment (180 μg, one time per week), and in one patient at the eighth week of Pegasys treatment (180 μg, one time per week). Pegasys treatment induced the development of ACLF in one patient, and pre-ACLF in two patients. Clinical characteristics at onset of severe acute exacerbation among three patients with chronic hepatitis B were shown in Table 2.

**Table 2:** Clinical characteristics at onset of severe acute exacerbation

	Case 1	Case 2	Case 3
Weeks of pegasys trreatment	2	8	2
HBeAg	+	_	_
Anti-HBe	_	+	+
HBV DNA (LC/ml)	5.74	6.23	4.10
WBC(10^9^/L)	3.82	2.95	4.24
Hb (g/L)	151	136	142
Plt(^x^10^9^/L)	85	55	64
ALT (lU/L)	1672	419	566
AST (lU/L)	1533	320	394
AST/ALT ratio	0.92	0.76	0.70
ALP (lU/L)	175	177	188
y-GTP(lU/L)	170	181	318
Total protein(g/L)	57.7	70.6	60.0
Albumin (g/L)	34.2	33.0	34.5
Total bilirubin(μmol/L)	289.3	179.6	180.6
Direct bilirubin (μmol/L)	182.5	146.6	96.8
Prothrombin activity (%)	35	83.3	88.1
AFP (ng/ml)	89	774.1	56.2
Creatine (μmol/L)	44.3	57	72.7
FT3 (pmol/L)	3.6	3.2	3.8
FT4 (pmol/L)	12.4	13.4	11.9
TSH (mlU/L)	1.3	0.9	1.2

HBeAg: hepatitis B e antigen; HBV: hepatitis B virus; LC/ml: Log copies/ml; WBC: white blood cell; Hb: hemoglobin; Plt: platelet; ALT: alanine aminotransferase; AST: asparate aminotransferase; ALP: alkaline phosphatase; γ-GTP: gamma glutamil transpeptidase; AFP: alpha fetoprotein; T3 and T4: thyroxin 3 and 4; TSH: thyroid-stimulating hormone.

### Clinical course and treatment

**Figure 1:**
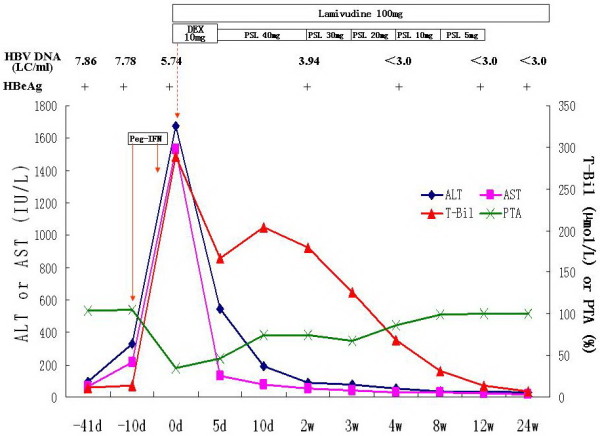
**Clinical course of case 1. Peg-IFN: pegylated interferon-α2a; LC/ml: Log**_**10**_**copies/ml; DEX: dexamethasone; PSL: prednisolone; ALT: alanine aminotransferase; AST: asparate aminotransferase; T-Bil: total bilirubin; PTA: prothrombin activity.**

Case 1 was tested for serum ALT being 96 IU/L, T-Bil 11.4 μmol/L and HBV DNA 7.86 Log_10_ copies/ml (LC/ml) on December 11 of 2008, but did not receive any antiviral treatment. After one month (January 11, 2009), he was tested for serum ALT being 333 IU/L, T-Bil 13.9 μmol/L and HBV DNA 7.78 LC/ml, and received antiviral treatment with Pegasys (180 μg, one time per week). He only felt slight fever (body temperature 38.1°C) and malaise at the first day of Pegasys treatment, and was not observed to be dark-coloured urine and icteric bulber conjunctiva during the first week Pegasys treatment. However, he complained of reduced appetite, vomiting and dark-coloured urine, and his bulber conjunctiva was observed to be icteric at the eighth day of Pegasys treatment. So, he was admitted to our hospital, and diagnosed to be ACLF based on his laboratory findings at the eleventh day of Pegasys treatment (Table 2). After admission, he was immediately treated with dexamethasone (10 mg/d, intravenously, for five days) and lamivudine (LMV, 100 mg/d). Serum ALT and T-Bil showed a decreasing tendency, and prothrombin activity (PTA) showed a increasing tendency, after which he started to receive a tapering-dose prednisolone therapy and antiviral treatment with LAM (Figure 1). The levels of serum ALT (35 IU/L), T-Bil (14.1 μmol/L) and PTA (100%) were normalized at 12 weeks treatment, after which he stoped to receive prednisolone therapy and continued to take LAM. He had undetectable level of serum HBV DNA (Real-time polymerase chain reaction assay,Amplicor HBV Monitor Test, Roche Diagnostics, Mannheim, Germany; detection limit of 1 × 10^3^ copies/mL) at 4 weeks LAM treatment, and sustained virologic response during LAM treatment. The loss of HBeAg was observed at 52 weeks LAM treatment, and HBeAg seroconversion at 64 weeks LAM treatment.

**Figure 2:**
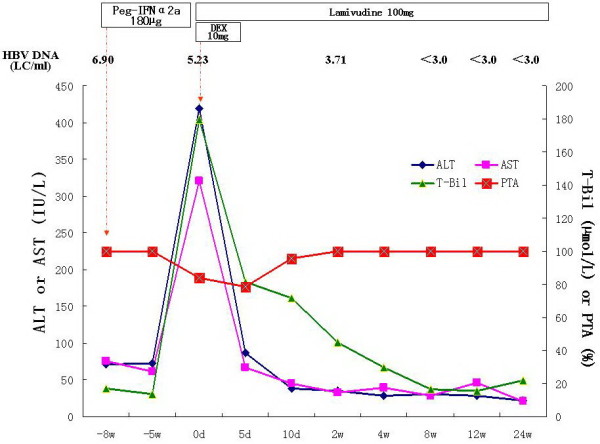
**Clinical course of case 2. Peg-IFNα2a: pegylated interferon-α2a; LC/ml: Log**_**10**_**copies/ml; DEX: dexamethasone; ALT: alanine aminotransferase; AST: asparate aminotransferase; T-Bil: total bilirubin; PTA: prothrombin activity.**

Case 2 started to complain of reduced appetite, headache, vomiting and dark-coloured urine after 8 weeks Pegasys treatment, and was admitted to our hospital. After admission, he was diagnosed to be pre-ACLF based on his laboratory findings (Table 2), and was immediately treated with dexamethasone (10 mg/d, intravenously, for five days) and LMV (100 mg/d). At 5 days of treatment, serum T-Bil was 81.4 μmol/L, and the decline extent of serum T-Bil is 54.7%, so dexamethasone therapy was stopped. After the cessation of dexamethasone therapy, the ALT and T-Bil still showed a decreasing tendency, and were normalized at 8 weeks treatment (Figure 2). He had undetectable level of serum HBV DNA at 8 weeks LAM treatment, and sustained virologic response during LAM treatment.

**Figure 3:**
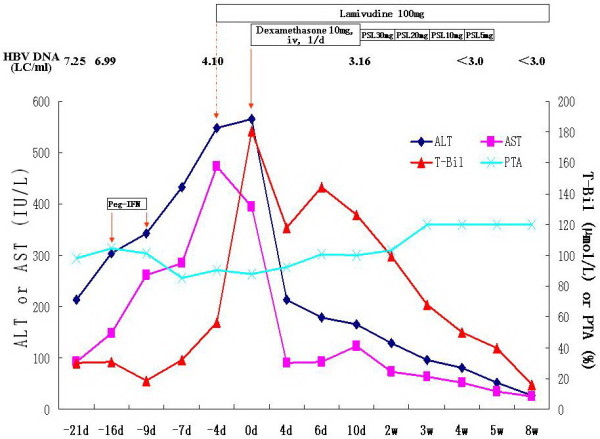
**Clinical course of case 3. Peg-IFN: pegylated interferon-α2a; LC/ml: Log**_**10**_**copies/ml; PSL: prednisolone; ALT: alanine aminotransferase; AST: asparate aminotransferase; T-Bil: total bilirubin; PTA: prothrombin activity.**

Case 3 was tested for serum ALT being 213 IU/L, T-Bil 30.2 μmol/L and HBV DNA 7.25 LC/ml on September 1 of 2010, and admitted to our hospital for antiviral therapy with Pegasys (180 μg, one time per week) because of suffering from chronic hepatitis B on September 6 of 2010 (Table 1). The changes of ALT, T-Bil and PTA were shown in Figure 3. Because of serum T-Bil being 56.4 μmol/L and ALT being 549 IU/L (normal < 42 IU/L) at 13^th^ day of Pegasys treatment, he started to receive LAM therapy instead of Pegasys. However, serum T-Bil was still rapidly increased after 4 days LAM treatment. He was diagnosed to be ACLF based on his worsened laboratory findings (Table 2), and immediately received a tapering-dose corticosteroid therapy (Figure 3). After corticosteroid therapy, the ALT and T-Bil showed a decreasing tendency, and were normalized at 8 weeks treatment (Figure 3). He had undetectable level of serum HBV DNA at 4 weeks LAM treatment, and sustained virologic response during LAM treatment.

Additionally, transfusion of magnesium isoglycyrrhizinate injection (200 mg/d) and reduced glutathione (1200 mg/d) were also given for 4 weeks among all three patients after being diagnosed to be ACLF or pre-ACLF.

## Consent section

The written informed consents were obtained from three patients before Pegasys and corticosteroid were administrated. Written informed consent was obtained from three patients for publication of this case report and any accompanying images. A copy of the written consent is available for review by the Editor-in-Chief of this journal."

## Discussion

IFN-α is able to increase the expression of HLA-I antigens on hepatocytes, which attracts T lymphocytes, with subsequent cytolytic and noncytolytic viral inactivation [7]. Acute exacerbation or ALT flare was usually observed among patients with chronic hepatitis B during IFN-αtreatment, and appeared to predict a successful outcome [2]. However, patients with severe acute exacerbation tend to have a higher risk for progression to ACLF with extremely high mortality [5,6]. Severe acute exacerbation or ACLF induced by standard interferon-α therapy had been reported to occur in few patients with chronic hepatitis B [3,4]. Pegylated interferon-α has been used to treat chronic hepatitis B and C for more than five years. Liver failure induced by pegylated interferon-α therapy had been reported in chronic hepatitis C [8]. But, no report showed that pegylated interferon-α therapy was able to induce severe acute exacerbation of chronic hepatitis B. Although severe acute exacerbation or ACLF during pegylated interferon-α is rarely occurred, but much attention should be paid by practicing physicians who take care of patients with chronic hepatitis B,because it is a life- threatening adverse effect. In the present cases, severe acute exacerbation of chronic hepatitis B occurred in two patients at the second week of Pegasys treatment, and in one patient at the eighth week of treatment.

Up to 30% of patients with chronic hepatitis B, included both HBeAg- positive and –negative patients, experience spontaneous reactivation of hepatitis every year. Severe acute exacerbation or ACLF may occur among a proportion of patients, and be associated with spontaneous HBe seroconversion[5]. However, we think that severe acute exacerbation or ACLF occurred in three patients described here, is closely associated with Pegasys therapy, but is not a progressive course of underlying hepatitis activity, based on the following reasons. Firstly, case 1 did not spontaneously progress into severe acute exacerbation or ACLF during one month follow-up without antiviral treatment, but progressed into ACLF after two-weeks treatment with Pegasys. Secondly, HBe seroconversion in case 1 was observed after 64 weeks of LAM treatment or severe acute exacerbation being diagnosed, indicated that severe acute exacerbation in case 1 was not associated with spontaneous seroconversion of HBeAg. Thirdly, only mild ALT elevation and normal T-Bil levels were observed in case 2 at the onset and after three weeks of Pegasys treatment. Forthly, severe acute exacerbation was not observed in case 3 during twelve days follow-up from five days of pretreatment to the end of first week Pegasys treatment, but was observed after the five days of second week Pegasys treatment. Additionally, no superinfection by HAV, HCV, HDV, HEV, CMV, EBV and HIV was detected in three patients. Autoimmune hepatitis (AIH), alcoholism and drugs other than Pegasys were also excluded in three patients.

The mortality is very high once ACLF or pre-ACLF develop in patient with severe acute exacerbation of chronic hepatitis B. No evidence showed that antiviral therapy with nucleoside analog (NA) was able to improve short-term mortality in patients with ACLF or pre-ACLF associated HBV infection [5,6]. Case 3 started to receive LAM therapy instead of Pegasys when he was diagnosed to be severe acute exacerbation at 13^th^ day of Pegasys treatment. Interestingly, serum T-Bil was still rapidly increased after 4 days LAM treatment, indicated that short-term antiviral treatment alone was not able to prevent the disease progression once severe acute exacerbation was diagnosed.

However, corticosteroid can rapidly inhibit excessive immune response and inflammatory reaction, and have been confirmed to be effective in treating patients with pre-ACLF and the early stage of ACLF [6,9]. Our data also showed that early intervention with corticosteroid and LAM was able to improve the liver function and prognosis of patients with ACLF and pre-ACLF. Our results also indicated that 5-days corticosteroid therapy was enough for rapid responders (the decline extent of serum T-Bil being ≥50% at 5 days), and a tapering-dose corticosteroid therapy was necessary for slow responders (<50%).

In summary, Pegasys therapy has a risk of inducing severe acute exacerbation including ACLF and pre-ACLF. Liver function should be tested once a week within 12 weeks of Pegasys therapy in order to find the risk of severe acute exacerbation as early as possible. Importantly, early combined intervention with corticosteroid and lamivudine should be introduced to prevent the disease progression and improve their prognosis once severe acute exacerbation was diagnosed.

## Competing interests

Financial competing interests

1. In the past five years, we did not received any reimbursements, fees, funding, or salary from any organization that may in any way gain or lose financially from the publication of this manuscript. There is not any organization financing this manuscript (including the article-processing charge).

2. We do not hold any stocks or shares in an organization that may in any way gain or lose financially from the publication of this manuscript, either now or in the future.

3. We are currently applying for any patents relating to the content of the manuscript. We did not receive reimbursements, fees, funding, or salary from any organization that holds or has applied for patents relating to the content of the manuscript.

4. We have not any other financial competing interests.

Non-financial competing interests

There are not any non-financial competing interests (political, personal, religious, ideological, academic, intellectual, commercial or any other) to declare in relation to this manuscript.

## Authors’contributions

Qing Mao collected the clinical data of one patient and drafted the manuscript, Hui-Yan Zhang collected the clinical data of one patient, Jian-Ping You collected the clinical data of one patient. Xu-Qing Zhang conceived of the study, participated in its design and performed the statistical analysis. All authors read and approved the final manuscript.
